# Compound heterozygous mutations in the *LTBP2* gene associated with microspherophakia in a Chinese patient: a case report and literature review

**DOI:** 10.1186/s12920-021-01080-0

**Published:** 2021-09-17

**Authors:** Manhua Xu, Kaiming Li, Weimin He

**Affiliations:** 1 Research Laboratory of Ophthalmology and Vision Sciences, State Key Laboratory of Biotherapy, West China Hospital of Sichuan University, Chengdu, 610000 Sichuan Province China; 2 Affiliated Hospital of Southwest Medical University, No.25, Taiping Street, Jiangyang District, Luzhou, 646000 Sichuan Province China

**Keywords:** Microspherophakia, *LTBP2*, Compound heterozygous mutations, Ectopia lentis, Secondary glaucoma

## Abstract

**Background:**

Microspherophakia (MSP, OMIM 251,750) is a rare inherited autosomal recessive eye disorder characterized by small spherically shaped lens. Several studies have indicated that the transforming growth factor-beta (TGF-beta) binding proteins(*LTBP2*) gene mutation is the predominant cause of MSP. In our study, novel compound heterozygous mutations in the *LTBP2* gene associated with MSP were reported, which was different from previous reported homozygous mutations.

**Case presentation:**

The proband was an 18‐year‐old male in Western China with bilateral MSP, accompanied by ectopia lentis, secondary glaucoma and blindness in both eyes. In our hospital, he received bilateral lens resection and trabeculectomy combined with peripheral iridotomy. Using next-generation sequencing (NGS)-based gene panel tests, we identified pathogenic mutations in the peripheral blood DNA sample from the proband: c.3614_3618dupCTGGC (exon24, NM_000428) and c.2819G > A (exon18, NM_000428). The presence of the novel compound heterozygous mutations in the *LTBP2* gene was linked with the development of MSP. Sanger sequencing confirmed the existence of one of the two variants in each parent respectively.

**Conclusion:**

Our results demonstrated a rare case of MSP phenotype associated with novel compound heterozygous mutations in the *LTBP2* gene using NGS technology.

## Background

Microspherophakia (MSP, OMIM 251,750) is a rare autosomal recessive ocular disorder characterized by an abnormally small spherical lens shape. Due to insufficient force generated by the hypoplastic suspensory ligaments the lens fails to form a normal oval shape [1]. MSP can cause ocular anomalies such as megalocornea, ectopia lentis and secondary glaucoma, and is related to hereditary systemic diseases such as Marfan syndrome (MFS) and Weill-Marchesani syndrome (WMS) [2, 3]. The homozygous mutation in latent transforming growth factor beta binding protein 2 (*LTBP2*) gene is considered the predominant cause for MSP [4]. In this study, we identified a novel *LTBP2* compound heterozygous mutation in a Chinese family through next-generation sequencing (NGS) technology. To the best of our knowledge, this case reported for the first time that MSP was associated with potentially causative *LTBP2* heterozygous mutations.

## Case presentation

### Case description

**Fig. 1:**
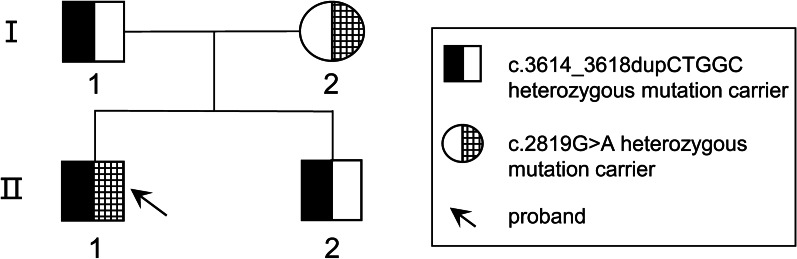
A two-generation family pedigree. The black arrow indicates the proband. Squares denote males. Circles denote females. Heterozygous individuals carrying either mutation are presented with half-filled shaded areas. The proband possessing compound heterozygous mutations is presented with both halves filled

**Fig. 2:**
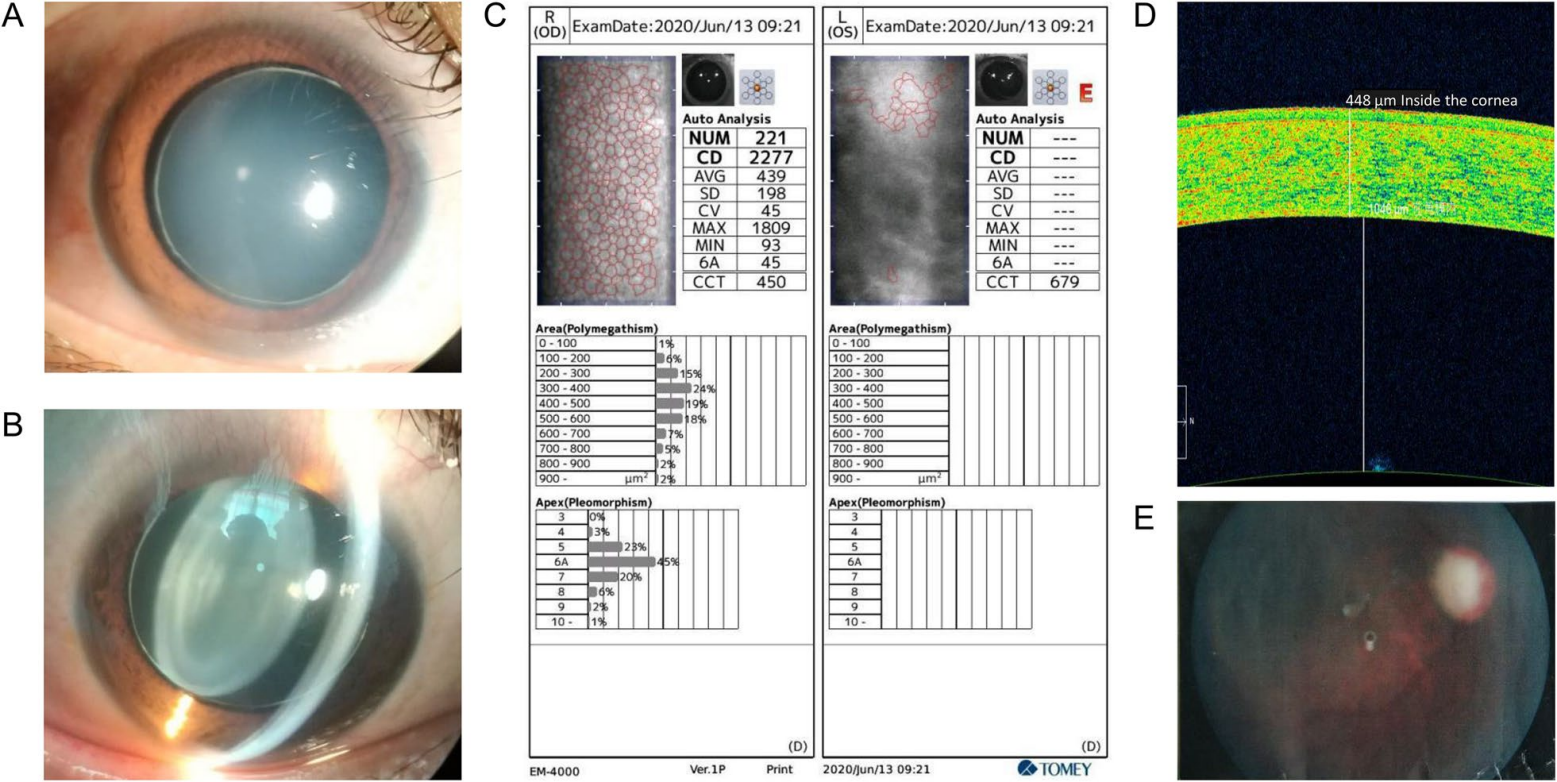
Clinical examination of the right eye. **a** and **b** Slit-lamp photography of the right eye showed normal cornea, small and spherical lens, large pupil, and stretched suspensory ligaments. **c** Microscopy images and endothelial cell characteristics of corneal endothelium in the right eye. No abnormalities were observed. NUM: number of cells; CD: Cell density; AVG: Average cell area; SD: standard deviation; CV: coefficient of variation; MAX: Maximum cell area; MIN: minimum cell area; 6A: Percentage of hexagonal cells; CCT: central corneal thickness. **d** Anterior-segment optical-coherence tomography (AS-OCT) in the right eye. CCT = 448 μm. **e** Posterior optical-coherence tomography (PS-OCT) revealed a pale optic disc in the right eye, with a cup-to-disc (C/D) value of 0.9

**Fig. 3:**
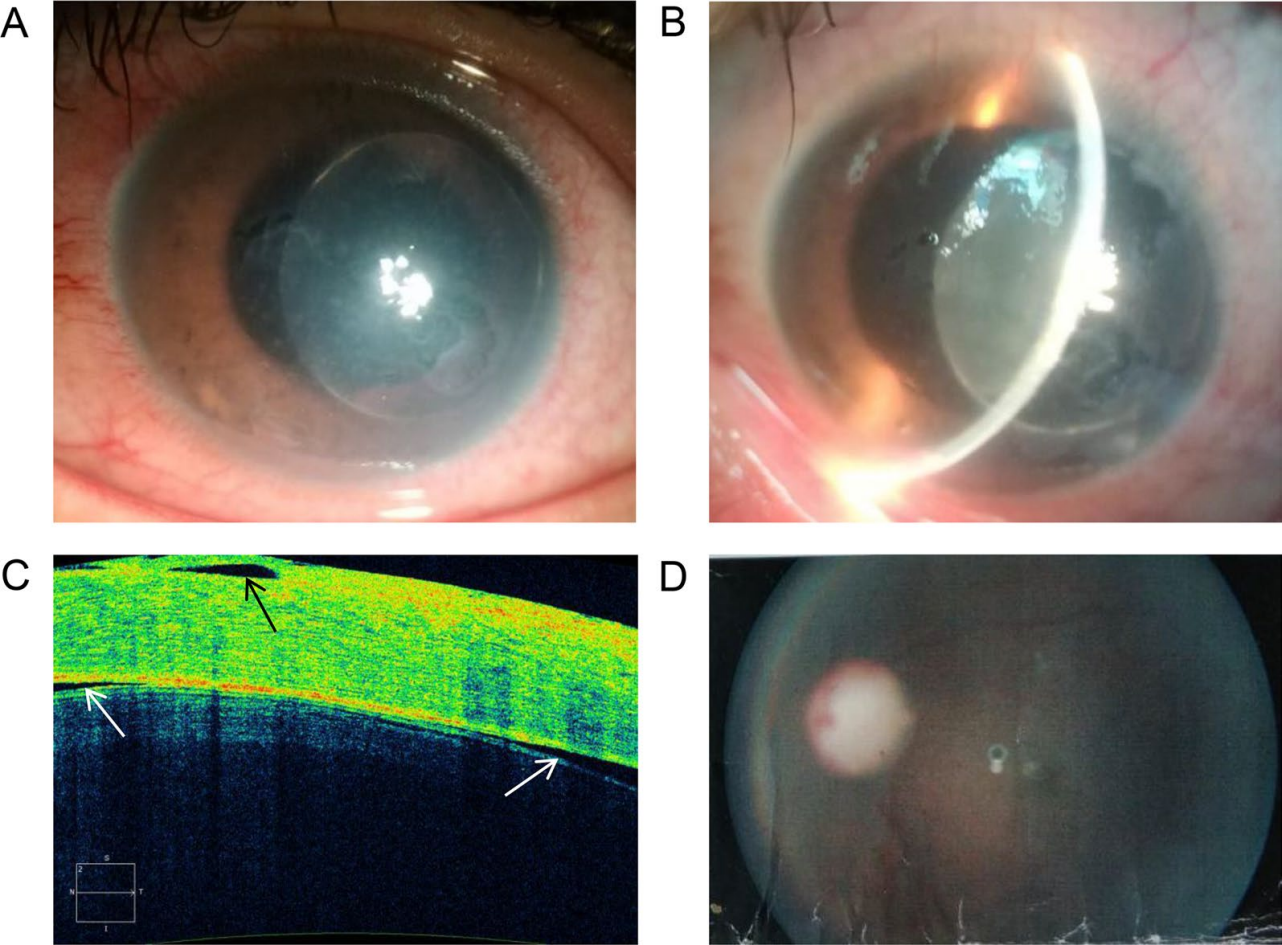
Clinical examination of the left eye. **a** and **b** Slit-lamp photography of the left eye showed corneal edema (**a**) and anteriorly dislocated lens (**b**). **c** AS-OCT revealed synechiae between the cornea and the lens. The black arrow indicates the subepithelial effusion. The white arrows indicate the lens anterior capsules. **d** PS-OCT revealed a pale optic disc in the left eye, with a C/D value of 1.0

**Fig. 4:**
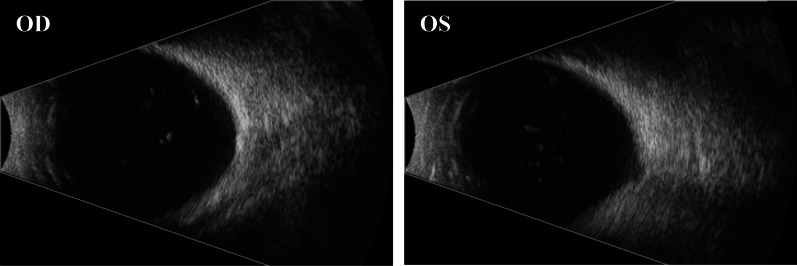
Binocular B-ultrasound examination. The prolongations of the axial axis were 28.73 mm in the right eye (left panel) and 28.38 mm in the left eye (right panel)

This study was approved by the Ethics Committee of Yanyuan People's Hospital (Xichang, Sichuan Province, China). Informed consent was obtained from each participant. The proband was an 18‐year‐old male from Southwest China (Fig. 1), born with blurred vision in both eyes. Six years ago, he experienced swollen pain in the left eye, accompanied by a headache on the left side and subsequent vision loss in a few months. Two years later, he had similar vision loss in the right eye. The medical history of the proband was unremarkable. He presented to our hospital with the complaint of long-term complete blindness, and severe pain in the left eye persistent in the past two months. Detailed interview revealed no systemic involvement, family history, trauma or surgery. An eye exam showed loss of light perception in binocular vision. Slit-lamp examination of the right eye showed no conjunctival hyperemia or edema. The right eye had a clear cornea with a normal diameter of 12 mm (Fig. 2a), a large round pupil (Φ = 7 mm) without light reflex, and a transparent, small spherical lens (Fig. 2b). The suspensory ligaments and the equator of the lens could be recognized through the dilated pupil under high magnification with a slit-lamp microscope, and the cell density of corneal endothelial cells was normal (2277 cells/mm^2^; Fig. 2c). Anterior segment optical coherence tomography (AS-OCT) showed thin central corneal thickness (448 μm) and narrow anterior chamber (1046 μm) (Fig. 2d). Posterior segment OCT (PS-OCT) revealed pale and atrophic optic disc (Fig. 2e). No abnormalities were found in the retina, but hyperemia and slight edema were seen in the conjunctiva. In left eye clinical examination, severe edema and opacity were observed throughout the cornea despite of normal corneal diameter (Fig. 3a), making it impossible to detect the corneal endothelial cell density. In addition, anterior chamber disappeared and the lens dislocated (Fig. 3b). AS-OCT revealed synechiae between the thickened cornea and the swollen lens (Fig. 3c). The pupil was found to be round and large (Φ = 7 mm) without reflex to light, and the fundus was invisible due to corneal opacity. PS-OCT revealed a pale and atrophic optic disc (Fig. 3d). High intraocular pressures (IOP) was seen in this case, with the right and left eye measured 47 mmHg and 50 mmHg, respectively. Binocular B-ultrasound imaging showed that the prolongations of the axial axis were 28.73 mm in the right eye and 28.38 mm in the left eye (Fig. 4).

No systemic abnormality was observed in this proband. The results of blood and metabolic tests (including liver and kidney function tests, homocysteine detection, plasma electrolyte test and contagion test) were all negative. Abdominal ultrasound, chest X-ray, brain CT and cardiac color Doppler ultrasound revealed no other abnormalities. No family members exhibited MSP phenotypes.

The proband was diagnosed with: 1. binocular MSP; 2. anterior lens subluxation of the left eye; 3. binocular secondary glaucoma; 4. corneal endothelial dysfunction of the left eye; and 5. binocular blindness. After hospitalization, the patient received binocular lens extraction and trabeculectomy combined with peripheral iridotomy to reduce intraocular pressure and ease the pain. After surgery, the proband still had no vision, but the pain disappeared.

### Genetic analysis

Genetic analyses were performed using the blood samples from the proband, his family members including parents and brother, and 100 ethnically matched healthy controls. Peripheral blood sample (5 mL) was collected from each participant into an EDTA-containing tube. Genomic DNA was isolated from peripheral blood lymphocytes using a DNA extraction kit (CWBio, Beijing, China) and sheared into 150-bp fragments (3 µg). The DNA library was constructed using the NEBNext DNA library prep reagent set (NEB, Ipswich, MA, USA). The target ophthalmological genes were captured using a GenCap capture kit (MyGenostics, Beijing, China) containing probes for 188 genes related to lens disease, iris disease, homocysteinosis, metabolic diseases and deafness. The DNA library was enriched by mixing 1 μg genomic DNA and the probes, followed by PCR amplification. NGS was performed on a DNBSEQ-T7RS sequencer (MGI, China) for paired read 150 bp (average sequencing depth: 1485.68; target area coverage: 10X: 99.93 20X: 99.87).

**Fig. 5:**
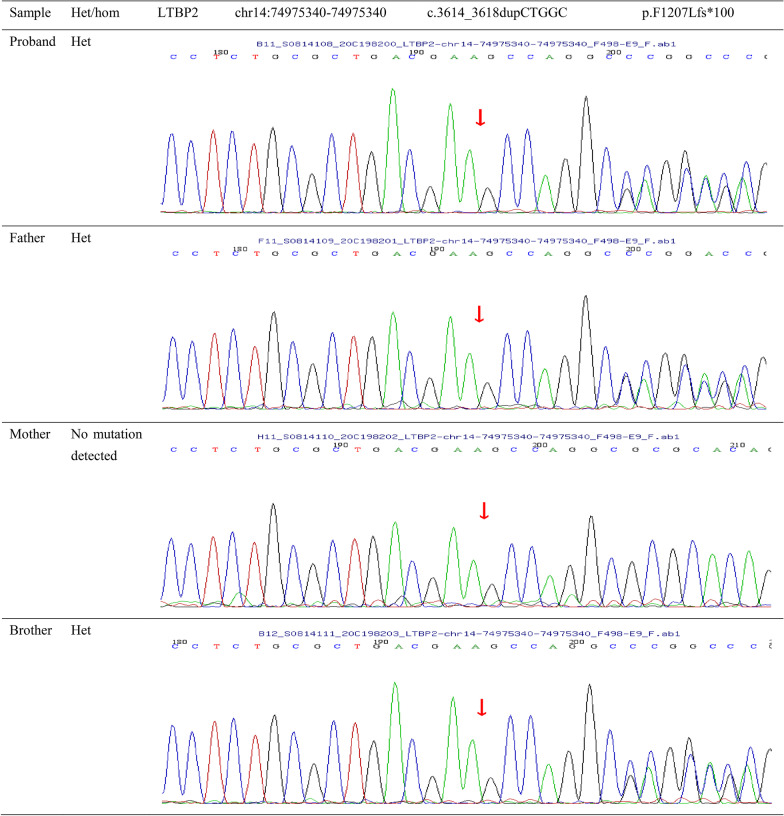
Pedigree analysis of c.3614_3618dupCTGGC(p.F1207Lfs*100) in *LTBP2*. Sanger sequencing identified a heterozygous c.3614_3618dupCTGGC (p.F1207Lfs*100) mutation in the LTBP2 gene of the proband, his father and the brother, but not his mother. The mutation is in exon 24 of LTBP2 gene. The red arrows indicate the start point of the mutation. Duplication of CTGGC at position 3614 may result in a phenylalanine-to-leucine substitution at phenylalanine-1207, followed by a stop codon at position + 99

**Fig. 6:**
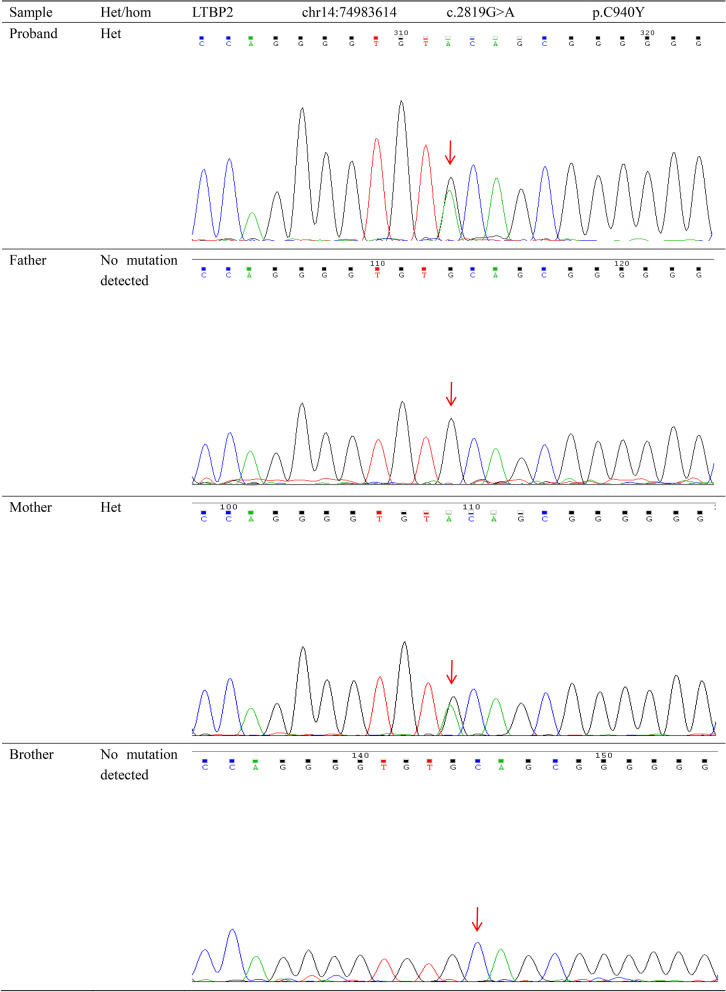
Pedigree analysis of c.2819G > A (p.C940Y) in *LTBP2*. Sanger sequencing identified a heterozygous c.2819G > A (p.C940Y) mutation in *LTBP2* gene of the proband and the mother, but not the father and brother. The mutation is in exon 18 of *LTBP2* gene. The red arrow indicates the mutation site. The G-to-A mutation at position 2819 may result in a cysteine-to-tyrosine substitution at position 940 in LTBP2 protein

**Fig. 7:**
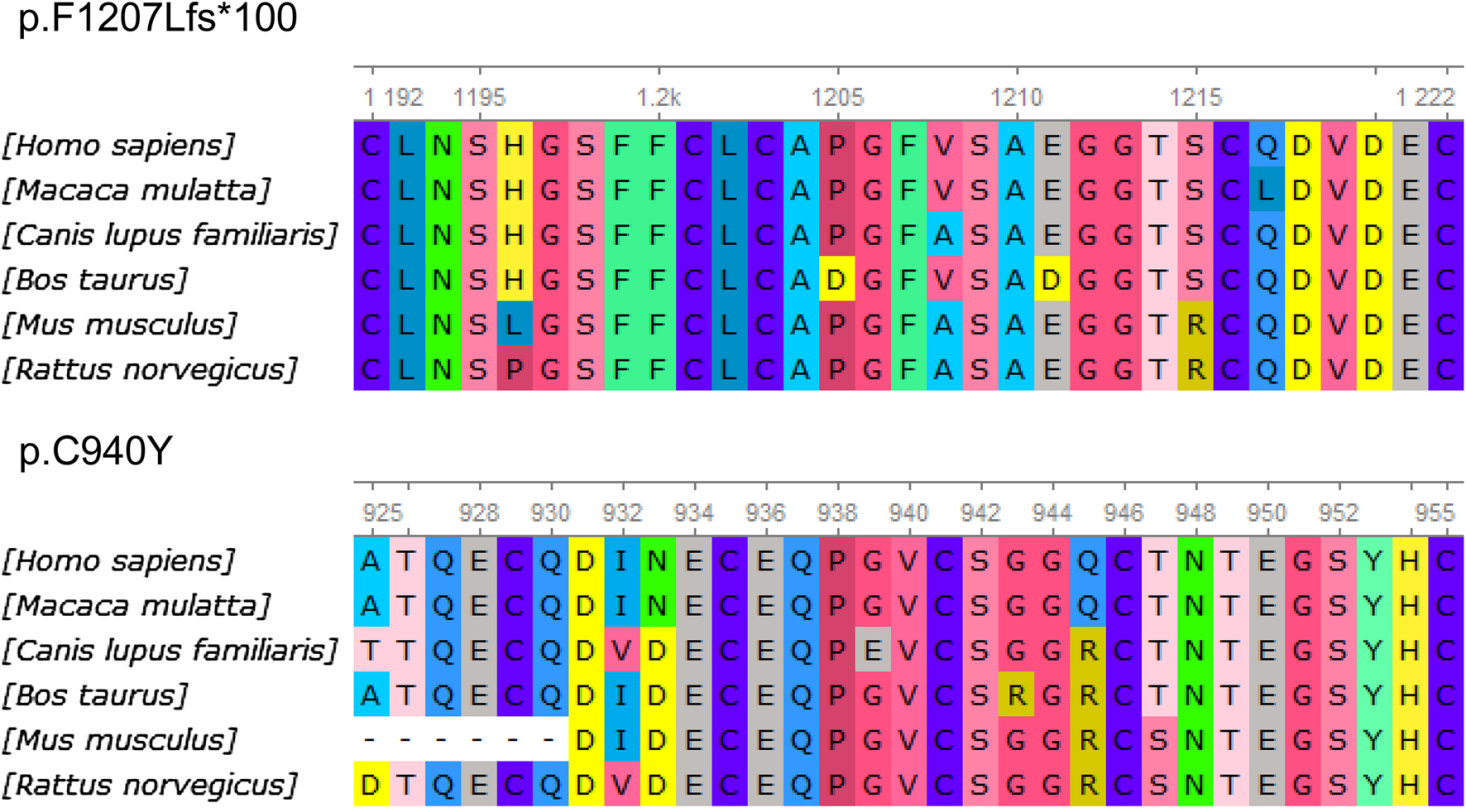
Amino acid sequence alignment of *LTBP2* from different mammalian species. Red arrows indicate phenylalanine-1207 and cysteine-940 that are highly conserved in these species

After removing the low-quality reads (< 80 bp), contamination and linkers from the raw data, the clean reads were mapped to the human reference genome hg19 using the BWA software. The single nucleotide variations (SNVs) as wells as inserts and deletions (INDEL) were identified using GATK software, followed by annotation using ANNOVAR software. The mutations with minor allele frequencies (MAF) less than 0.02 were excluded. The pathogenicity and conservatism of missense mutations were predicted using SIFT, PolyPhen-2, MutationTaster, GERP++ and REVEL. The pathogenicity of splice site changes was analyzed using SPIDEX. Suspicious mutations were assessed according to the variation interpretation guideline of American College of Medical Genetics and Genomics (ACMG). Finally, six suspicious mutations were identified. After excluding four mutations in *ABHD12*, *PXDN*, *ERCC6* and *RECQL4* that are unrelated to MSP, two mutations in MSP-related *LTBP2* were selected, including a frameshift mutation c.3614_3618dupCTGGC (exon24, NM_000428) (chr14:74975340–74975340) (Fig. 5) and a missense mutation c.2819G > A (exon18, NM_000428) (chr14:74983614) (Fig. 6). Protein prediction revealed that the two mutations might cause coding changes in LTBP2 protein, yielding p.F1207Lfs * 100 and p.C940Y, respectively. These two sites are highly conserved in mammalian species (Fig. 7).

The two *LTBP2* mutations were found not to be reported in the 1000 Genomes Project, EXAC, gnomAD and dbSNP databases. MutationTaster predicted both mutations as “Disease causing”. ACMG also predicted the c.3614_3618dupCTGGC variant as “pathogenic”. The c.2819G > A variant was predicted as “deleterious” by SIFT, indicating that the mutation might affect protein function. PolyPhen-2 score of c.2819G > A was 0.999 (> cut-off value 0.95), suggesting that the mutation might influence protein structure and function. GERP +  + score of c.2819G > A mutation was 4.79 (> cut-off value 2), suggesting that it was highly conserved and might exert great effects on protein structure and function. Besides, the REVEL score of c.2819G > A was 0.74, which demonstrated that the mutation was deleterious. Thus, the two *LTBP2* variants were considered pathogenic.

The DNA samples of the proband, his family members, and 100 healthy controls were subjected to Sanger sequencing to validate the variants. The proband’s father and brother were found to harbor a heterozygous c. 3614_3618dupCTGGC mutation, the mother harbored a heterozygous c.2819G > A mutation, and the proband carried both mutations (Figs. 5, 6). The two *LTBP2* genetic variants were not found in healthy controls.

## Discussion and conclusion

The proband had a typical clinical phenotype and pathogenic progress of MSP. With increasing age, the suspensory ligaments of the lens stretched and broke, spontaneously subluxed, leading to corneal endothelial dysfunction and secondary glaucoma. High IOP contributed to optic atrophy, causing bilateral blindness of the patient.

Studies have shown that various microfibril-associated gene mutations were related to lens diseases.

*Ectopia lentis* Ectopia lentis is a common feature of several syndromic disorders such as MFS, homocystinuria and MSP. Isolated ectopia lentis is frequently caused by genetic alterations, mainly *ADAMTSL4* mutations [7–9]. *ADAMTSL10/17*, fibrillin-1 (*FBN1*), *CBS*, *CPAMD8* and *LEPREL1* gene variations are also associated with ectopia lentis [10–12].

*Marfan syndrome (MFS)* MFS is an autosomal dominant connective tissue disorder characterized by tall and thin stature, long arms, legs, fingers and toes, and sometimes accompanied by cardiovascular disorders [13]. Lens subluxation, myopia and blurred vision are common manifestations in patients with MFS. Approximately 90% of MFS cases are caused by mutations in the *FBN1* gene [14, 15].

*Weill-Marchesani syndrome (WMS)* WMS is a rare inherited autosomal recessive or dominant disorder of the connective tissue, characterized by short stature, short fingers, brachydactyly, joint stiffness, sometimes heart defects, and eye anomalies such as MSP, lens subluxation, severe myopia and glaucoma. *FBN1*, *LTBP2*, *ADAMTS10*, *ADAMTS17* gene mutations have been reported to be associated with the occurrence and development of WMS [16–19].

*Homocystinuria (HCU)* Classic HCU due to cystathionine β-synthase (CBS) deficiency is a genetic disorder of sulfur metabolism following an autosomal recessive inheritance. Its clinical manifestations include mental retardation, intraocular lens dislocation, skeletal abnormalities, and a tendency to thromboembolic episodes. *CBS* gene mutations are the predominant cause of HCU [20–22].

*MSP* So far, there have been few reports on MSP pathogenic genes, and *LTBP2* (OMIM 602,091) is the only known causative gene for MSP [4]. *LTBP2*, located in 14q24.3, contains 35 exons and encodes a protein composed of 1821amino acids. The function of LTBP2 protein still remains unclear. Some studies have shown that *LTBP2* may play a critical role in eye development. Narooie-Nejad et al. [23] reported that human eyes had high *LTBP2* expression levels in the trabecular meshwork, ciliary process, Descemet membrane and lens capsule, but minimal expressions in the corneal stroma, sclera and iris. *LTBP2* is structurally and functionally homologous to *FBN1* and *FBN2*. Additionally, *LTBP2* is essential for the development of the ciliary zonule microfibrils that are required for assembling elastic fibers and suspending lenses. *LTBP2* deficiency causes lens luxation due to ciliary zonule fragmentation [4, 24, 25]. Ocular diseases associated with *LTBP2* include WMS, MSP, megalocornea, and ectopia lentis [26–29]. Kumar et al. [30] found that a homozygous duplication mutation (c.5446dupC) in exon 36 of *LTBP2* could lead to isolated MSP. Alías et al. [28] detected the insertion of an adenine (c.5439_5440insA) in exon 36 of *LTBP2* in a Spanish family with MSP. Shahzadi et al. [31] presented a genetic linkage between MSP and *LTBP2* locus in a large consanguineous Pakistani family.

In our study, we identified two *LTBP2* variants in the pedigree. The frameshift mutation c.3614_3618dupCTGGC changed the reading frame starting from phenylalanine-1207 (phenylalanine-to-leucine), and the new reading frame stopped at the100^th^ codon downstream of phenylalanine-1207. The missense mutation c.2819G > A caused a cysteine-to-tyrosine substitution in LTBP2 protein. MutationTaster and ACMG predicted c.3614_3618dupCTGGC as “Disease causing” and “pathogenic”, respectively. SIFT identified c.2819G > A as “deleterious”. MutationTaster assessed c.2819G > A as “disease causing”. The PolyPhen-2 score of c.2819G > A was 0.999, greater than the cut-off value of 0.95, suggesting that this variant might affect the structure and function of LTBP2 protein. The GERP +  + score of c.2819G > A was 4.79, greater than the cut-off value of 2. This finding suggested that this site was highly conserved, and the variant was highly likely to impair the function of LTBP2 protein. Besides, the REVEL score of c.2819G > A was 0.74, indicating that the mutation was deleterious. These results collectively suggested that the two *LTBP2* variants were pathogenic. Furthermore, by searching the Conserved Domain Database of NCBI, we found that the two sites of the *LTBP2* gene were highly conserved in different species, including *homo sapiens*, *mus musculusand*, *macaca fascicularis*, *canis lupus*, *bos taurus*, *mus musculus*, and *rattus norvegicus*, suggesting that mutations in these sites may significantly affect the function of LTBP2 protein. Importantly, neither of these two mutations has been reported in 1000 Genomes Project, EXAC, gnomAD and dbSNP databases.

We further verified the two *LTBP2* genetic variants in the pedigree and ethnically matched healthy controls using Sanger sequencing (Fig. 1). Neither mutation was present in the healthy controls. The father and brother of the proband carried c.3614_3618dupCTGGC mutation, and the mother carried c.2819G > A mutation. Compound heterozygous mutations containing both the paternal and maternal variants were detected in the proband [30]. Only the the proband presented MSP-related clinical phenotypes. Thus, we concluded that the *LTBP2* compound heterozygous mutations were potentially causative genetic variants for MSP.

In summary, we identified novel compound heterozygous mutations of *LTBP2* gene (c.3614_3618dupCTGG and c.2819G > A) that were associated with MSP risk. This finding highlighted the significance of *LTBP2* gene in the etiology of MSP, expanded the mutation spectrum of *LTBP2*, providing a new reference for further research on MSP.

## Data Availability

The datasets generated and/or analysed during the current study are available in the NCBI BioProject database under the accession number PRJNA753875 (https://www.ncbi.nlm.nih.gov/Traces/study/?acc=PRJNA753875). Public databases used in this study included Human reference genome (GRCH37/hg19) (https://www.ncbi.nlm.nih.gov/assembly/GCF_000001405.13/), 1000 genomes database (http://www.1000genomes.org/), EXAC (http://exac.broadinstitute.org/), gnomAD (http://gnomad.broadinstitute.org/about), and dbSNP (http://www.ncbi.nlm.nih.gov/projects/SNP/).

## Abbreviations

MSP: Microspherophakia
MFS: Marfan syndrome
WMS: Weill–Marchesani syndrome
LTBP2: Latent transforming growth factor beta Binding Protein 2
NGS: Next-generation sequencing
AS-OCT: Anterior segment optical coherence tomography
PS-OCT: Posterior segment OCT
IOP: Intraocular pressure
SNV: Single nucleotide variation
INDEL: Inserts and deletion
MAF: Minor allele frequency
ACMG: American College of Medical Genetics and Genomics
FBN1: Fibrillin-1
HCU: Homocystinuria
CBS: Cystathionine β-synthase
